# Fetal biometric and Doppler measurements following abdominal radical trachelectomy in the second trimester of the pregnancy

**DOI:** 10.1186/s12884-022-04671-6

**Published:** 2022-04-20

**Authors:** Eiri Shima, Mina Itsukaichi, Kosuke Yoshihara, Tatsuya Ishiguro, Kazufumi Haino, Koji Nishino, Nobumichi Nishikawa, Koji Nishijima, Takayuki Enomoto

**Affiliations:** Department of Obstetrics and Gynecology, Niigata University Graduate School of Medical and Dental Sciences, 1-757 Asahimachi-dori, Niigata, 951-8510 Japan

**Keywords:** Abdominal radical trachelectomy, Cervical cancer, Pregnancy, Fetal growth

## Abstract

**Background:**

Our previous study demonstrated the safety and effectiveness of abdominal radical trachelectomy during pregnancy but did not focus on the fetus. This study aimed to clarify the influence of abdominal radical trachelectomy performed during pregnancy on the fetus.

**Methods:**

Eight cervical cancer patients who underwent abdominal radical trachelectomy at our hospital between February 2013 and August 2020 were enrolled in this study. To assess the peri- and postoperative influence on the fetus, we performed fetal heart monitoring at 30-min intervals during abdominal radical trachelectomy and calculated the estimated fetal body weight and resistance indexes of the middle cerebral artery and umbilical artery from postsurgery until delivery.

**Results:**

Four out of eight patients had preterm birth due to chorioamnionitis in one case and consideration of the recurrent risk of cervical cancer in three cases. Fetal heart monitoring during abdominal radical trachelectomy revealed deceleration just once in one case but no abnormal findings in the other cases. In all cases, the fetal growth after abdominal radical trachelectomy was normal until delivery. No abnormal Doppler findings were detected in the middle cerebral artery or umbilical artery.

**Conclusion:**

Our findings clarified that abdominal radical trachelectomy performed for the treatment of early-stage cervical cancer during pregnancy has no obvious influence on fetal growth. Next, it is necessary to evaluate the growth and development of children delivered from mothers who have undergone abdominal radical trachelectomy during pregnancy.

**Supplementary Information:**

The online version contains supplementary material available at 10.1186/s12884-022-04671-6.

## Background

Uterine cervical cancer is the most frequently diagnosed malignant tumor in women of childbearing age and pregnant women worldwide [1]. The incidence of late marriage and late childbearing is increasing in Japan, as in many other developed countries. Hence, the number of women who aspire to undergo fertility-preserving treatment, even if they have been diagnosed with cervical cancer, is increasing. In Japan, the human papillomavirus (HPV) vaccination rate among a specific age range of girls intended to receive HPV vaccination is less than 1% and the cervical cancer screening rate at all ages is 30–40%, which are remarkably low percentages compared with those of other countries [2, 3]. Under these circumstances, patients who are diagnosed with invasive cervical cancer during their pregnancies are not as rare in Japan as they are elsewhere.

Radical trachelectomy (RT) for the treatment of early-stage uterine cervical cancer has been widely established within the past decade, and accumulated obstetrical and gynecological outcome data support its efficacy and safety [4–8]. RT has become a valid option to preserve the fertility of patients with early-stage cervical cancer. However, the treatment of cervical cancer during pregnancy remains a major challenge. RT during pregnancy (RT-DP) is still a controversial treatment option because of limited related data, but there are dozens of case reports on abdominal radical trachelectomy (ART) performed on pregnant women to save both the mother and baby at the same time [9–11]. Most of these studies, including our previous report [12], support the effectiveness of RT even during pregnancy.

In terms of obstetric management, RT shortens the uterine cervix length and may make pregnant women more susceptible to intrauterine infection. Therefore, pregnancy after RT occurs with apparent high-risk for preterm labor. Several studies have discussed the timing of delivery in post-RT pregnancy but have not focused on the fetus during such pregnancies. Indeed, to date, there is no evidence regarding intrauterine fetal growth or newborn long-term outcomes in post-RT pregnancy. The changes in blood flow to the uterus after RT may affect intrauterine fetal growth and well-being. In addition, anesthesia and direct surgical manipulation of the uterus containing the fetus during RT may affect intrauterine fetal growth and well-being.

Therefore, our aim in this study was to clarify the influence of abdominal radical trachelectomy during pregnancy (ART-DP) on the fetus. This is the first retrospective large cohort study to assess the safety and effectiveness of ART-DP with a focus on the fetus and newborn.

## Methods

This was a retrospective observational study of fetuses whose mothers received ART for the treatment of cervical cancer in their second trimester between October 2013 and August 2020 at our hospital. This study was approved by the Institutional Ethics Review Board at Niigata University (No. 2017–0265). The ART-DP indication requires the following five criteria at our institute: 1) strong desire to preserve ongoing pregnancy, 2) surgery can be performed at 15–17 weeks of gestation, 3) International Federation of Gynecology and Obstetrics (FIGO) stage IB1 (tumor size < 2 cm), 4) no lymph node metastasis suspected preoperatively, and 5) squamous cell carcinoma or adenocarcinoma. All patients received information about the procedure, related complications, daily restriction during pregnancy after the surgery, and other therapeutic options in an unbiased and non-coercive manner. During surgery, the fetal heart rate was measured every 30 min to check for bradycardia (Additional file 1). After ART-DP, intravenous tocolysis with administration of isoxsuprine hydrochloride before 16 weeks’ gestational age (GA) and ritodrine hydrochloride after 16–0 GA was started routinely until postoperative uterine contractions and lower abdominal pain disappeared. If an abnormal increase in uterine contractions was observed, tocolysis was initiated by administrating ritodrine and/or magnesium sulfate (MgSO_4_) [13–15]. Moreover, a progesterone (250 mg) intramuscular injection was given routinely per week until delivery if there were no adverse effects. All patients were hospitalized after ART-DP until their deliveries. Vaginal washings and cervical canal length measurements were performed regularly. The timing of termination was selected based on an overall assessment of the gestational age, the recurrent risk of cancer, and the patient and fetal conditions.

Transabdominal ultrasound examination was performed to assess fetal growth and well-being at least once a week. The estimated fetal body weight (EFBW), amniotic fluid depth, and Doppler velocimetry of the middle cerebral artery (MCA) and umbilical artery were measured during each examination. Body weight and height at birth were used to assess the general health status of the newborns. The status of appropriate for date (AFD), light for date (LFD), or small for date (SFD) was classified based on the neonatal anthropometric charts [16]. Child development was investigated by interviewing the patient at the periodic checkup. All management was conducted by experienced gynecological oncologists and perinatal professionals.

## Results

A total of eight patients who underwent ART-DP in our hospital were included in this study, and the patient characteristics are shown in Tables 1 and 2. The patients’ average age at the date of surgery was 32.8 (27–44) years. The rate of preterm birth was 50% (4/8). Four out of eight patients had preterm births; this was due to chorioamnionitis in Case #1, and scheduled termination was considered for a recurrent risk of cervical cancer in Cases #3, #4, and #6. Regarding postoperative complications, infectious diseases occurred in Cases #1 and #2, probably due to infection ascending to the uterus. Case #1 was complicated with threatened preterm delivery, chorioamnionitis (CAM), and premature rupture of membranes. Case #2 developed a pelvic abscess on the 38th postoperative day. Antibiotic intravenous treatment worked effectively and the abscess disappeared. The rapid premature rupture of membranes (PROM) test (Check PROM®; OHKURA Pharmaceutical Co. Ltd., Kyoto, Japan) yielded a positive result on the 38th postoperative day, but the test yielded a negative result after the abscess was cured. The amniotic fluid had been normal amount, and amniotic leak did not persist, therefore high PROM had suspected the cause of the positive result of the PROM test [17]. Afterward, the closure of the neo cervical external os during ART-DP was performed in the following cases (Cases #3–#8) to prevent ascending infection (Additional file 2). Complications related to ascending infection were not observed in the aforementioned cases after the addition of this procedure.

**Table 1:** Patient characteristics and perioperative parameters of ART-DP

Case No	Age	Stage	Histology	GA at surgery	Uterine arteries	Placenta location (MRI^**a**^)	Progesterone 250 mg im per week	IV tocolysis after surgery	Treatment for threatened preterm delivery	Postsurgical complications
1	36	IB1	SCC	15–0	Bil. preserved	Fundus to upper right wall	Until delivery	27 days	32-5GA ~ Ritodrine, 33-3GA ~ Ritodrine + MgSO4	CAM
2	33	IB1	SCC	17–0	Rt. preserved	Fundus to middle anterior wall	Until delivery	13 days	23-0GA ~ Ritodrine + MgSO4, 26–5 ~ 28-5GA Ritodrine	Pelvic abscess
3	27	IB1	SCC	15–2	Bil. preserved	Rt. middle to low posterior wall	Until delivery	5 days	–	None
4	30	IB1	SCC	15–6	Not preserved	Lt. middle to low posterior wall	Until delivery	8 days	–	None
5	27	IB1	AD	15–1	Bil. preserved	Upper middle posterior wall	Until delivery	13 days	–	None
6	33	IB1	SCC	17–0	Rt. preserved	Fundus to middle anterior wall	Until delivery	12 days	–	None
7	32	IB1	SCC	15–5	Bil. preserved	Rt. upper anterior wall	Only 2 weeks	9 days	–	None
8	44	IB1	SCC	16–4	Bil. preserved	Fundus to posterior wall	Until delivery	9 days	25-6GA ~ Ritodrine	None

*GA* Gestational age, *MRI* Magnetic Resonance Imaging, *im* intramuscular injection, *SCC* Squamous cell carcinoma, *AD* Adenocarcinoma, *Bil* bilateral, *Rt* Right, *Lt* Left, *CAM* Chorioamnionitis

^a^MRI was performed 1–4 weeks before ART-DP

**Table 2:** Perinatal parameters and child development

Case No	Abnormal pregnancy	Preterm birth reason	GA at Cesarean delivery	Birth weight (g)	Birth height (cm)	Neonatal anthropometric	Children’s growth and development (Months following)
1	Threatened preterm delivery	CAM	33-5GA	1820	42	AFD	Normal (90 M)
2	Preterm PROM	–	37-3GA	2572	47	AFD	Normal (89 M)
3	None	Termination (LVSI +)	30-4GA	1462	39	AFD	Normal (81 M)
4	None	Termination (Surgical margin +)	33-4GA	2164	46	AFD	Language development delay (71 M)
5	None	–	37-1GA	2585	46	AFD	Normal (64 M)
6	None	Termination (Surgical margin +)	33-0GA	1742	42	AFD	Normal (53 M)
7	None	–	37-3GA	2996	52	AFD	Normal (40 M)
8	Threatened preterm delivery	–	37-4GA	2294	47	AFD	Normal (9 M)

*CAM* Chorioamnionitis, *PROM* Premature rupture of the membranes, *LVSI* Lymphovascular invasion, *AFD* appropriate for date compared with the normal range in the Japanese population

**Fig. 1:**
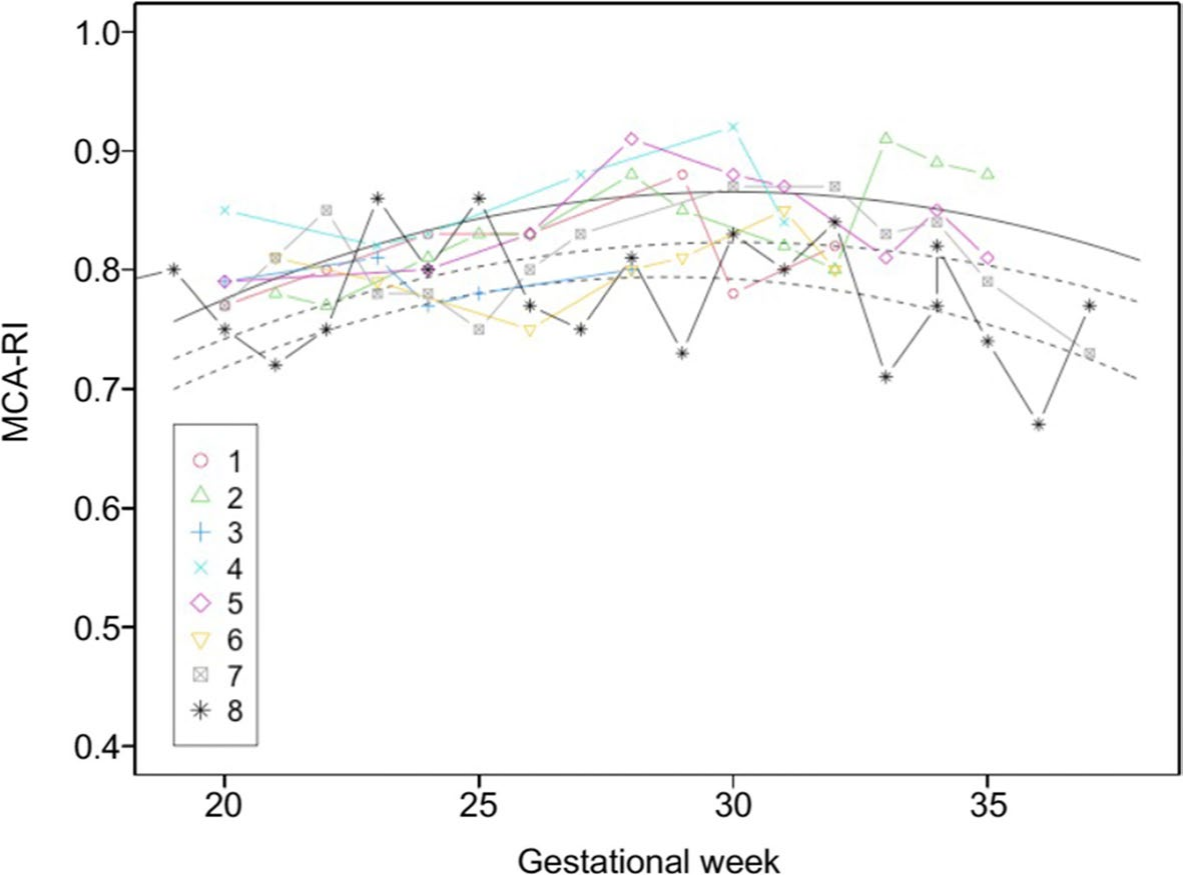
Changes in middle cerebral artery resistance index (MCA-RI) during pregnancy. MCA-RI measurement was performed every week. Each plot represents each value of MCA-RI. The solid line shows the average MCA-RI in each gestational week and the two dotted lines indicate the 10th percentile to 90th percentile. Each plot number corresponds to our case number

**Fig. 2:**
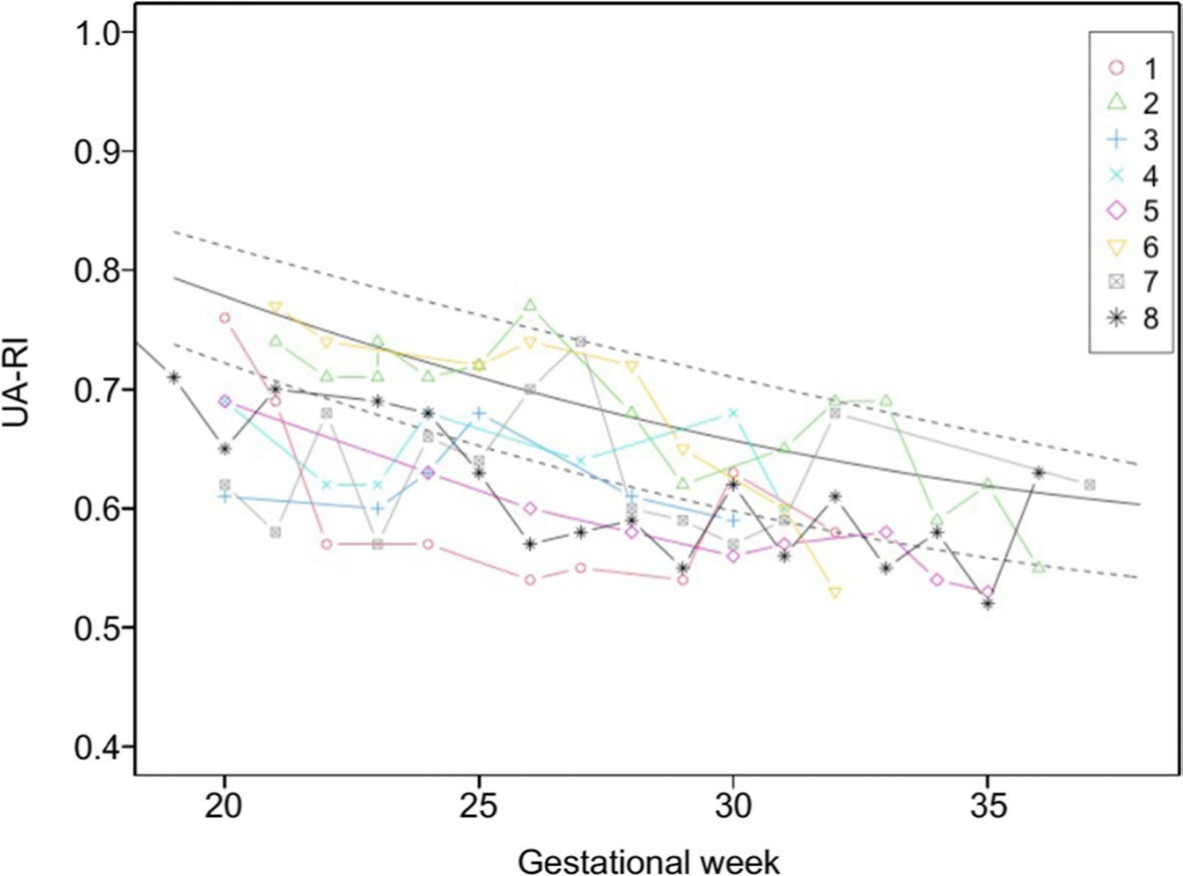
Changes in umbilical artery resistance index (UA-RI). Each plot represents each value of UA-RI. The solid line shows the average UA-RI in each gestational week and the two dotted lines indicate the 10th percentile to 90th percentile. Each plot number corresponds to our case number

**Fig. 3:**
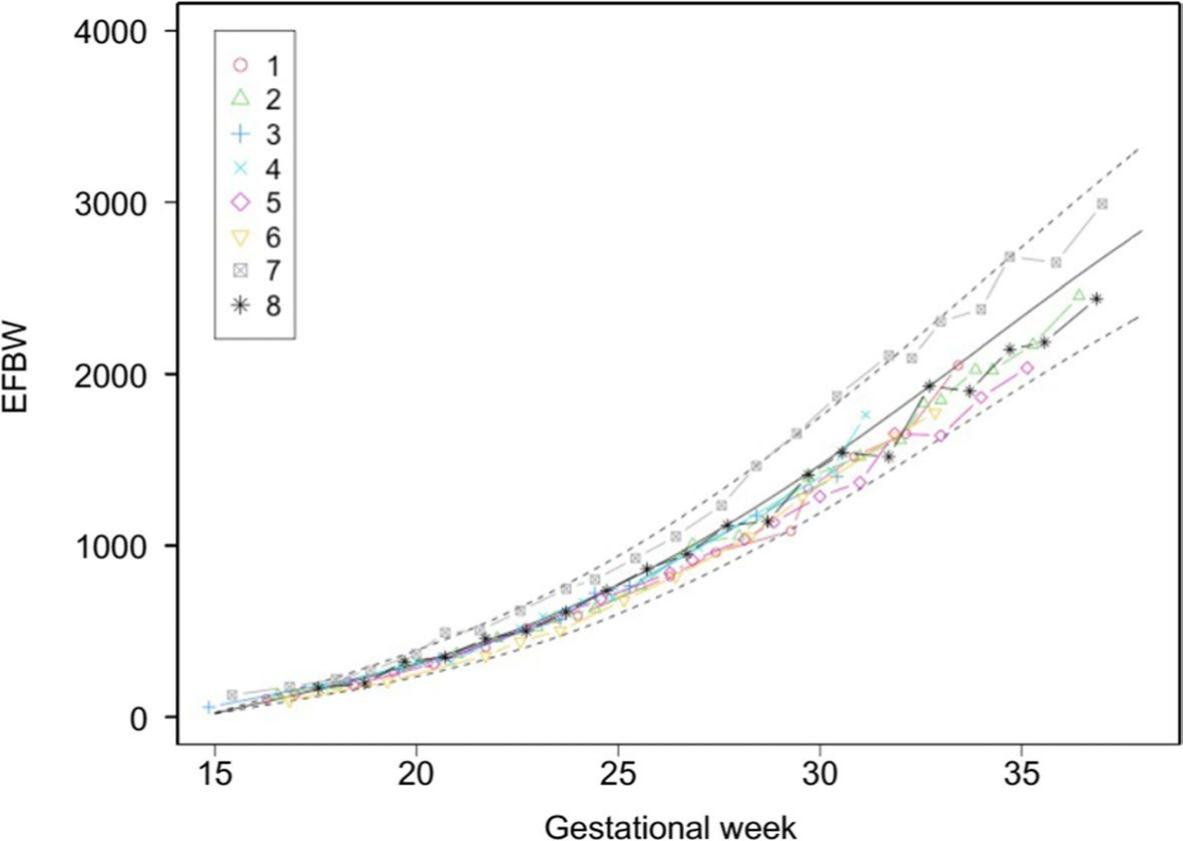
Changes of estimated fetal body weight (EFBW). Each dot represents EFBW for each gestational date. The solid line shows the average EFBW of the Japanese population in each gestational week. The zone between the two dotted lines is − 1.5 to 1.5 standard deviations. Each plot number corresponds to our case number

Preservation of the uterine arteries bilaterally succeeded in five cases, unilateral preservation succeeded in two cases, and the uterine arteries were not preserved in Case #4. Fetal heart monitoring during ART-DP showed a short period of fetal bradycardia just once in Case #7 but was not observed in the other cases. There was no apparent causality between fetal bradycardia and preserved uterine arteries or the location of the placenta. Doppler flows of the middle cerebral artery and umbilical artery did not show brain-sparing effects (Figs. 1 and 2), and EFBW was within the normal range for all fetuses (Fig. 3). All of the newborns were classified in the range of AFD. Although the mean follow-up period of 68 months was insufficient to evaluate the infants’ long-term outcomes, their growth remained in the normal range. In terms of development, a delay in language development was suspected in Case #4 who was born at 33 weeks of gestation, but no other developmental abnormalities have been suspected in the rest of the cases from periodic medical checkup.

## Discussion

In the treatment of pregnant women with cervical cancer, not only oncologic outcomes but also obstetric, neonatal, and pediatric outcomes such as fetal and newborn growth, development, and long-term health should be considered. A systematic review of ART-DP demonstrated that the fetal loss rate, PROM rate, and preterm birth rate were 21.1, 14.3, and 47%, respectively [18]. However, fetal growth and development have not been substantially discussed in the previous literature to date [18]. Therefore, this study focused on the influences of ART-DP on subsequent fetal growth and development.

First, we considered the influence of surgery itself on pregnancy. In general, the influence of surgery such as ovarian cystectomy and appendectomy during pregnancy has been shown to be low [19, 20]. Vujic et al. concluded that non-obstetric surgery during pregnancy could be carried out safely without obstetrical complications [21]. A recent review demonstrated that anesthetic agents commonly used during pregnancy were not associated with teratogenic effects in clinical doses [22]. However, ART-DP procedures might be related to ongoing pregnancy outcomes in terms of longer operation time and more blood loss compared with other surgeries performed during pregnancy. In addition, a shortened uterine cervix might increase the risk of PROM, CAM, and preterm delivery, as has been observed in pregnancies after conization [23]. In the present study, preterm delivery caused by CAM occurred in Case #1. After the addition of uterine cervix external os closure (Additional file 2), we were able to prevent ascending infection in all cases from Case #3 to Case #8. No complications related to the closure of cervical external os have been observed to date. Blood flow from the parametrium and paracolpium stops after ART-DP, and even blood flow from the uterine artery might be disrupted if bilateral uterine arteries cannot be preserved during radical trachelectomy. Insufficient uteroplacental blood supply could cause inadequate placental formation and asymmetrical fetal growth restriction, as frequently experienced in hypertensive disorders of pregnancy [24, 25]. However, contrary to expectations, all infants born after ART-DP were classified as AFD. Corresponding to this result, EFBW remained within the normal range, and abnormal Doppler flow was not observed in any fetus. The physical and mental development of most of the infants was also normal within the observation period. A delay of language development was suspected in Case #4, who was born at 33 weeks of gestation. Because delayed language development is well known as one of the complications seen in prematurely born children, it is unclear whether this complication was related to the operative procedure itself. Indeed, no apparent problems regarding growth and development were observed in four cases who were born at 37 weeks of gestation. The most recent literature review about ART-DP reported that the miscarriage rate after ART-DP was 19% and that most miscarriages occurred shortly after the operation [26]. A suspected reason for these miscarriages was insufficient blood flow because of the ligation of both uterine arteries, as only hypoxic changes of the placenta were observed during autopsy.

Neoadjuvant chemotherapy (NAC) is another treatment option for pregnant women with early-stage cervical cancer. As NAC has been administered during pregnancy to patients with various types of cancers, such as breast cancer [27], there are more data available regarding the influence of NAC on fetal and perinatal outcomes than that of ART-DP [28] Schwab et al. noted that chemotherapy during the second and third trimesters is considered relatively safe but increases the risk of preeclampsia and fetal growth restriction [29]. They noted the preterm birth rate was 48, and 12% of those births spontaneously occurred. Focusing on cervical cancer, Ilancheran asserted that NAC during pregnancy was a viable option and that fetal well-being was not compromised [30]. However, Song et al. reported that although 77 out of 88 cases, in which NAC treatment for cervical cancer occurred during pregnancy, involved completely healthy neonates, neonates in the remaining cases experienced complications including respiratory syndrome disorder, mild serum creatinine elevation, anemia, intraventricular hemorrhage, bilateral perceived hearing loss, hypotension, hypoglycemia, supraventricular tachycardia, and erythema [31]. In the long-term follow-up of those neonates, retroperitoneal embryonal rhabdomyosarcoma occurred in one case at the age of 5 and acute myeloid leukemia occurred in one case at the age of 22 months. It is difficult to prove the relationship between cancer occurrence in children and exposure to anticancer agents during the second and third trimesters. However, compared with the incidence rates of cancer in children aged 0–14 years [32], 2 out of 88 cases seems to be a relatively high incidence of cancer. It is necessary to consider the possibility of secondary cancer from exposure to anticancer agents during the second and third trimesters. In addition, the response rate to chemotherapy for cervical cancer should be considered [33, 34]. If the cancer progresses or spreads, patients may lose the chance to undergo surgery despite the presence of a resectable lesion at the time of diagnosis.

ART-DP is not a standard treatment for early-stage cervical cancer during pregnancy, and only limited data are available in the literature. In addition, ART-DP is performed at a limited number of institutes due to the difficulty of ART-DP procedure and the complications of postsurgical management. International Network of Cancer, Infertility, and Pregnancy (INCIP) recommends simple trachelectomy or NAC in early-stage cervical cancer because ART-DP has a high rate of obstetrical and surgical complications [35, 36]. However, ART-DP is one of the important treatment options for pregnancy-associated cervical cancer because some patients were successfully treated only by ART-DP.

## Conclusions

For pregnant women with invasive cervical cancer, the advantages and disadvantages of each treatment modality are essential to make the decision. Although the number of cases was small, our findings clarified that there was no obvious influence of ART-DP for pregnancy-associated cervical cancer on fetal growth and newborn development. After ART-DP, all fetuses were able to avoid exposure to anticancer agents. To establish the safety and effectiveness of ART-DP for pregnancy-associated cervical cancer, we need to continue investigating perinatal outcomes and children’s growth and development after ART-DP.

## Supplementary Information

**Additional file 1: Figure 1.** Image of fetal heart monitoring during surgery. During trachelectomy, the fetal heart rate was measured every 30 min to check for bradycardia.

**Additional file 2: Figure 2.** Images of cervical cerclage of neo cervix and closure of neo external os. Left panel shows the image after trachelectomy. Right panel demonstrated cervical cerclage and os closure.

## Data Availability

All data analysed during this study are included in this published article.

## Abbreviations

HPV: Human papillomavirus
RT: Radical trachelectomy
RT-DP: Radical trachelectomy during pregnancy
ART: Abdominal radical trachelectomy
ART-DP: Abdominal radical trachelectomy during pregnancy
FIGO: International Federation of Gynecology and Obstetrics
GA: Gestational age
MgSO_4_: Magnesium sulfate
EFBW: Estimated fetal body weight
MCA: Middle cerebral artery
AFD: Appropriate for date
LFD: Light for date
SFD: Small for date
CAM: Chorioamnionitis
PROM: Rapid premature rupture of membranes
NAC: Neoadjuvant chemotherapy
