# Supplementary material for: Fetal biometric and Doppler measurements following abdominal radical trachelectomy in the second trimester of the pregnancy
Source: BMC Pregnancy Childbirth. 2022 Apr 20;22:343. doi: 10.1186/s12884-022-04671-6 (PMC9022245; doi:10.1186/s12884-022-04671-6)
Supplement: Supplementary file 1 — Additional file 1: Figure 1. Image of fetal heart monitoring during surgery. During trachelectomy, the fetal heart rate was measured every 30 min to check for bradycardia. [file 12884_2022_4671_MOESM1_ESM.pdf]

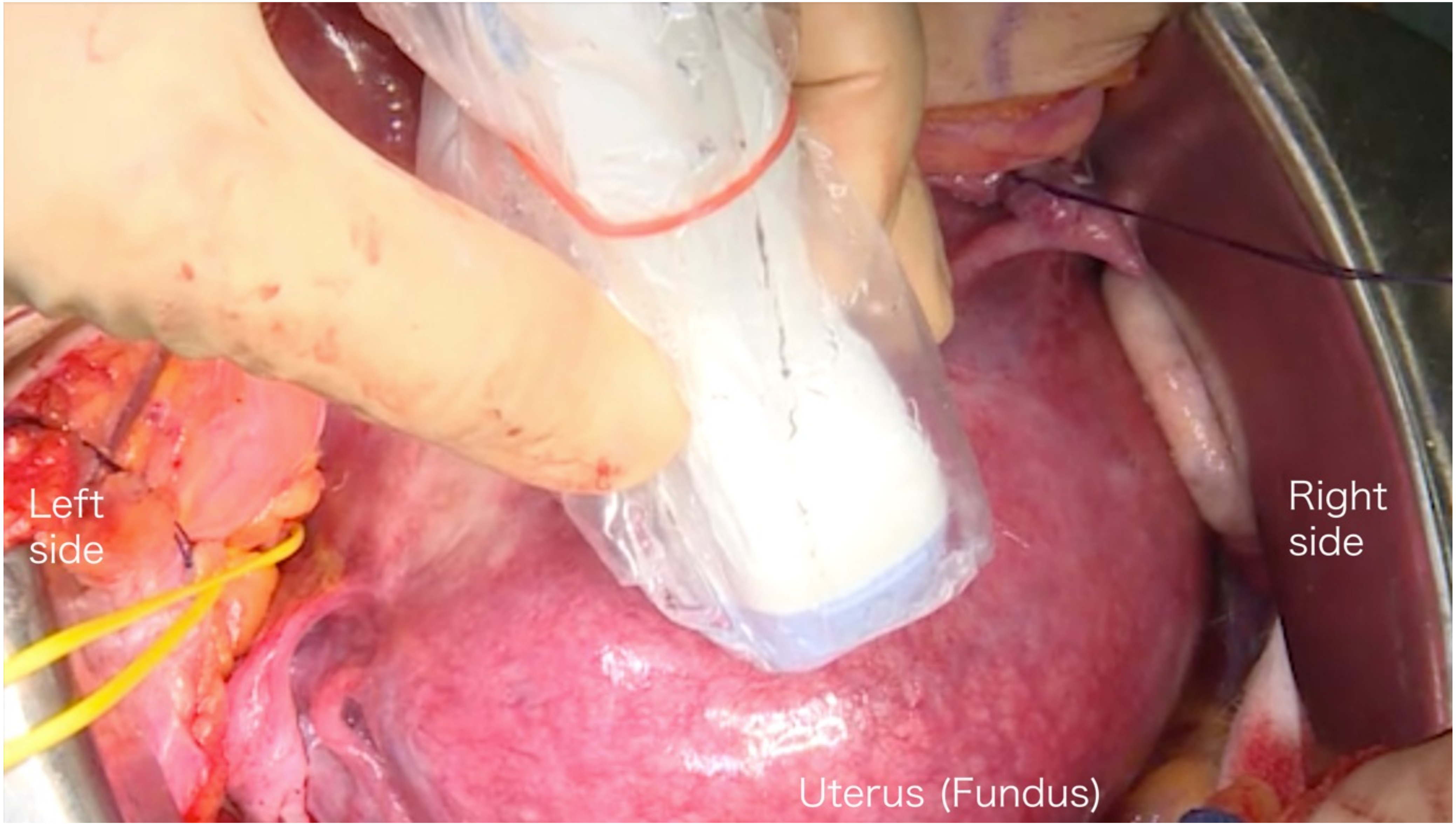

Additional file 1 Figure 1. Image of fetal heart monitoring during surgery
During trachelectomy, the fetal heart rate was measured every 30 minutes to check for bradycardia.
