# Supplementary material for: Fetal biometric and Doppler measurements following abdominal radical trachelectomy in the second trimester of the pregnancy
Source: BMC Pregnancy Childbirth. 2022 Apr 20;22:343. doi: 10.1186/s12884-022-04671-6 (PMC9022245; doi:10.1186/s12884-022-04671-6)
Supplement: Supplementary file 2 — Additional file 2: Figure 2. Images of cervical cerclage of neo cervix and closure of neo external os. Left panel shows the image after trachelectomy. Right panel demonstrated cervical cerclage and os closure. [file 12884_2022_4671_MOESM2_ESM.pdf]

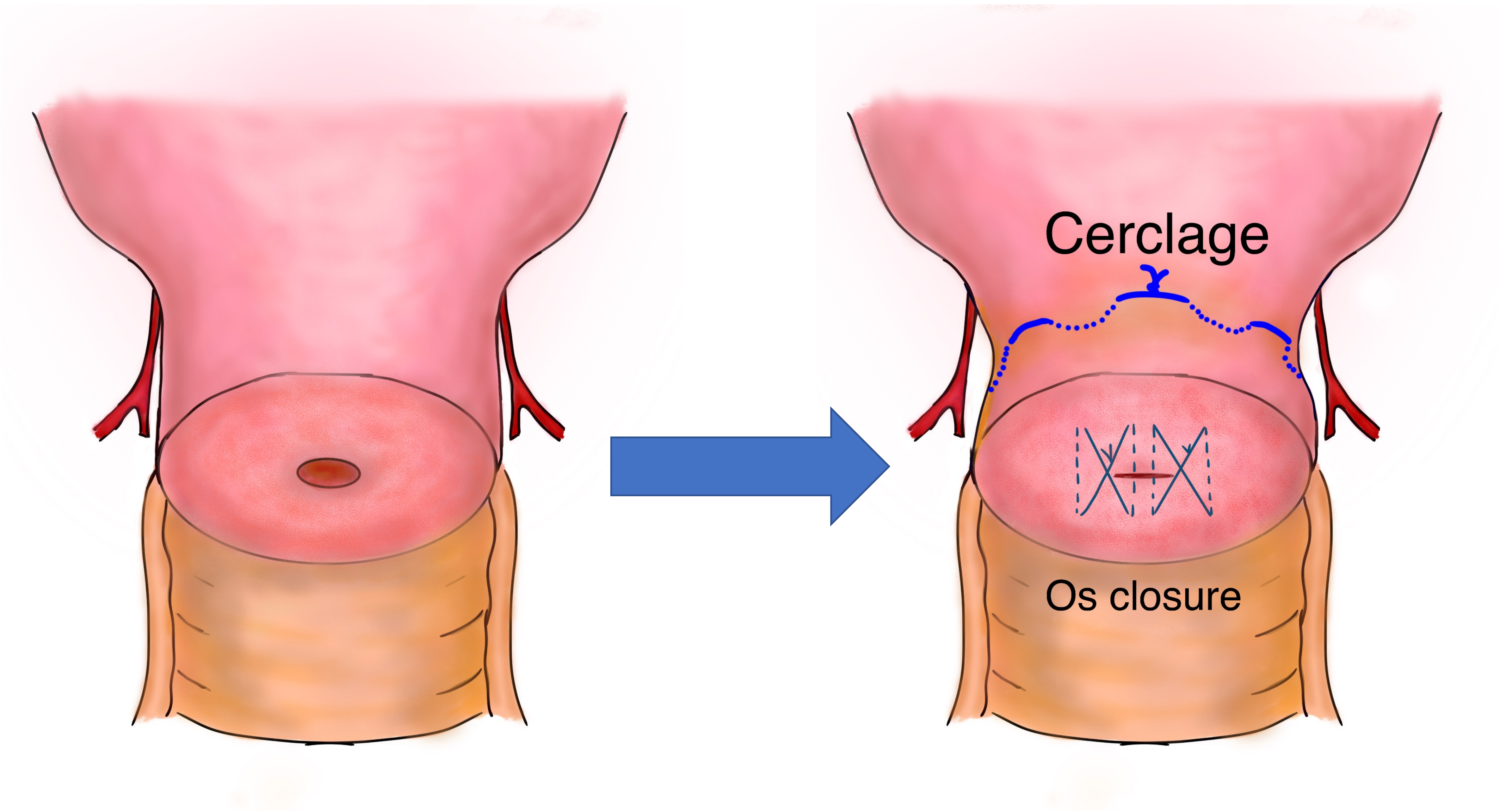

Additional file 2 Figure 2. Images of cervical cerclage of neo cervix and closure of neo external os

Left panel shows the image after trachelectomy. Right panel demonstrated cervical cerclage and os closure.
